# Structure bioinformatics of eight human ATP synthase F_0_ subunits and their AlphaFold3-predicted reduced-hydrophobicity QTY analogues

**DOI:** 10.1371/journal.pone.0352911

**Published:** 2026-10-23

**Authors:** Ziyu Wang, Edward Chen, Shuguang Zhang

**Affiliations:** 1 Deerfield Academy, Deerfield, Massachusetts, United States of America; 2 Media Lab, Massachusetts Institute of Technology, Cambridge, Massachusetts, United States of America; UP Diliman: University of the Philippines Diliman, PHILIPPINES

## Abstract

Human mitochondrial ATP synthase is an essential rotary motor enzyme that produces most of the cellular ATP through oxidative phosphorylation. Its membrane-embedded F_0_ sector contains highly hydrophobic transmembrane subunits that are challenging to study in aqueous environments without detergents. We applied the QTY code to eight human ATP synthase F_0_ subunits: ATP6, ATP8, ATPK, ATP68, ATPMK, AT5G1, AT5G2, and AT5G3. Hydrophobic amino acids leucine (L), isoleucine (I), valine (V), and phenylalanine (F) in transmembrane regions were systematically replaced with hydrophilic glutamine (Q), threonine (T), and tyrosine (Y). Four native subunits with available CryoEM structures from human ATP synthase (PDB: 8H9S) were superposed with their AlphaFold3-predicted QTY analogues. For subunits without complete CryoEM structures, AlphaFold3-predicted native structures were superposed with their AlphaFold3-predicted QTY analogues. Additional structural analyses were included, and key structural and functional regions, including ATP68 and the ATP6–c-ring interface, were modeled. The native ATP synthase F_0_ subunits superposed well with their respective QTY analogues. For the CryoEM-native comparisons, RMSD values ranged from 0.565 Å to 6.952 Å. For the AlphaFold3-native comparisons of subunits without CryoEM structures, RMSD values ranged from 0.204Å to 0.297Å. Considering all eight subunits, despite substantial QTY substitutions in the transmembrane regions, ranging from 38.89% to 50.79%, the QTY analogues retained similar overall folds, molecular weights, and isoelectric points. Hydrophobic surface analysis showed that the QTY analogues had reduced hydrophobic surfaces compared with their native counterparts. These structural bioinformatics studies suggest that the QTY code is applicable to ATP synthase F_0_ subunits to generate more hydrophilic, water-soluble analogues while preserving overall structural similarity. These results extend the application of the QTY code to the membrane-embedded F_0_ sector of ATP synthase and provide a foundation for future experimental studies for these QTY analogues expressed, purified, and evaluated for self-assembly.

## Introduction

Mitochondrial ATP synthase is one of the most conserved and essential motor enzymes in the human body. It is the final step of oxidative phosphorylation in aerobic respiration, producing most of the adenosine triphosphate (ATP) that cells use as energy [1–4]. ATP synthase plays key roles in both catalytic and transport mechanisms. Human ATP synthase is constructed with 29 subunits of 18 types, organized into membrane-intrinsic and membrane-extrinsic domains, linked by a central peripheral stalk. As such, the enzyme is a dual rotary motor built up of different subunits: the soluble catalytic F1 sector, where ATP is synthesized, and the membrane-embedded F_0_ sector, where proton translocation is converted into rotary motion.

The rotary mechanism comprises two elements: a rotor and a stator. The rotor includes the F_0_ c-ring and the central stalk, which rotates during proton translocation; the stator includes ATP6 and the peripheral stalk, which stabilize the catalytic F1 head against co-rotation [3,4].

The two sectors are coupled molecularly so that proton flow through the F_0_ sector drives conformational changes in F1 that synthesize ATP from adenosine diphosphate (ADP) and inorganic phosphate [1–5]. Together, these sectors couple proton flow down the electrochemical gradient across the inner mitochondrial membrane and drive ATP production with great efficiency. Because ATP synthase is a key component in cellular energy metabolism, even slight alterations to its structure could damage its assembly and coupling efficiency, causing severe physiological consequences and mitochondrial disease [1,3,6,7].

Within this complex, the F_0_ sector is significant for housing the proton-conducting motor and being the most hydrophobic part of the enzymes [2–5]. In humans, the F_0_ sector is composed of the c-ring together with subunit ATP6, ATP8, and several associated membrane and peripheral stalk subunits to stabilize the rotor architecture [3,8–10]. The F_0_ sector couples the movement of protons down their concentration gradient to the rotation of its c-ring, producing mechanical torque that is passed through the central stalk to the F1 sector. The F_0_ sector takes its name from its sensitivity to antibiotic oligomycin, which can bind to c-subunits to block proton flow, stopping rotation and ATP synthesis [8,11]. The F_0_ sector is suitable for structural analysis, since it’s highly membrane-embedded, yet its functions rely on precise structural positioning, subunit interactions, and tight packing between hydrophobic helices [3,5,8,11].

Our study focuses on eight ATP synthase F_0_ subunits. The abbreviated manuscript-specific protein labels used in this paper are ATP6, ATP8, ATPK, ATP68, ATPMK, AT5G1, AT5G2, and AT5G3. Official protein names, conventional subunit designations, gene symbols, and UniProt accessions are defined below and listed as part of Table 1.

**Table 1:** Standard nomenclature names, structural sources, tissue expression, and representative medical relevance of the eight human ATP synthase F_0_ subunits examined in this study.

Name	Gene symbol	UniProt accession	Conventional subunit	Structure, (Å PDB ID)	Tissue expression	Medical relevance
ATP6	*MT-ATP6*	P00846	Subunit a	CryoEM (2.53 Å, 8H9S)	Ubiquitous	Mitochondrial disease
ATP8	*MT-ATP8*	P03928	Subunit 8	CryoEM (2.53 Å, 8H9S)	Heart-enriched	Cardiomyopathy
ATPK	*ATP5MF*	P56134	Subunit f	CryoEM (2.53 Å, 8H9S)	Ubiquitous	Complex V deficiency
ATP6	*ATP5MJ*	P56378	Subunit j/6.8 PL	CryoEM (2.53 Å, 8H9S)	Skeletal muscle	ATP synthase assembly
ATP	*ATP5MK*	Q96IX5	Subunit k	AlphaFold3 predicted native structure	Ubiquitous	Complex V deficiency
AT5G	*ATP5MC1*	P05496	C-subunit1, isoform 1	AlphaFold3 predicted native structure	Ubiquitous	Oxidative phosphorylation
AT5G	*ATP5MC2*	Q06055	C-subunit2 isoform 2	AlphaFold3 predicted native structure	Ubiquitous	Oxidative phosphorylation
AT5G	*ATP5MC3*	P48201	C-subunit3, isoform 3	AlphaFold3 predicted native structure	Ubiquitous	Neuromuscular disease

The manuscript-specific labels, gene symbols, UniProt accessions, conventional subunit designations, and structural source used for native-protein analysis are listed for ATP6, ATP8, ATPK, ATP68, ATPMK, AT5G1, AT5G2, and AT5G3. Tissue expression and medical relevance are representative and not exhaustive. Gene symbols are italicized.

ATP6, officially ATP synthase F_0_ complex subunit a and encoded by mitochondrial gene MT-ATP6 (UniProt accession P00846), is the central mechanical component among the F_0_ subunits in this study. It is highly hydrophobic, forming the core stator adjacent to the rotating c-ring and providing the offset proton half-channels that allow protons to enter and leave the membrane domain during catalysis [3–5,12]. It helps generate the two offset proton half-channels required for proton entry, release, and generation of rotary movement [3,12]. Many pathogenic variants in MT-ATP6 can disrupt proton movement, a/c-ring coupling, or ATP synthesis rate [13–15].

ATP8, officially ATP synthase F_0_ complex subunit 8 and encoded by mitochondrial gene MT-ATP8 (UniProt accession P03928), is smaller and less involved in proton transfer, though studies have shown that it contributes to stabilization of ATP6 and the surrounding membrane domain during biogenesis of the complex. Interestingly, ATP6 and ATP8 are encoded in overlapping mitochondrial genes [8,10,16]. Clinical studies have shown that pathogenic MT-ATP8 variants can contribute to mitochondrial diseases, although their precise clinical interpretation remains challenging because they are less studied, may be influenced by mitochondrial heteroplasmy, and are infrequently reported. ATP8 is thought to contribute more to structural support than direct catalysis [16].

ATP68, officially ATP synthase F_0_ complex subunit j, mitochondrial, or 6.8 kDa mitochondrial proteolipid protein (6.8PL), encoded by the gene ATP5MJ (UniProt accession P56378), is another F_0_-associated subunit [8,9]. Its function has been less defined historically, though recent studies have shown its use in locking ATP6 and ATP8 into their membrane assembly. It also helps dimerize the monomer complex via interactions between j subunits. Studies have shown that removal of ATP68 hindered cell growth and a partial loss of ATP6 and ATP8 [8,17].

ATPK, officially ATP synthase F_0_ complex subunit f, mitochondrial, encoded by the gene ATP5MF (UniProt accession P56134), belongs to the supernumerary membrane region that helps maintain the structure of ATP synthase. Subunits like these occupy peripheral positions around the membrane motor, building up the membrane domain of the peripheral stalk. They contribute to local packing and maintain a wedge-shaped membrane, potentially helping with c-ring rotation [3,5,8–10]. ATPK has been shown to assist in dimer stability, though its decrease has shown little effect on ATP synthetic capabilities or the amount of other subunits [18].

ATPMK, officially ATP synthase F_0_ complex subunit k, mitochondrial, encoded by the gene ATP5MK (UniProt accession Q96IX5), acts in a similar capacity as ATPK, appearing in the peripheral stalk of ATP synthase. It’s also been classified as a diabetes-associated protein in insulin-sensitive tissue (DAPIT) [3,5,8–10]. Similar to ATPK, ATPMK promotes the structural stability of ATP synthase. Previous conclusions have classified ATPMK as a dispensable protein for the core functions of ATP synthase. However, recent studies have shown that suppression can cause a loss of ATP synthase population in the mitochondria and destabilization in the dimer structure. [19–21].

The three remaining proteins are AT5G1, AT5G2, and AT5G3, officially ATP synthase F_0_ complex subunits C1, C2, and C3, mitochondrial, encoded by genes *ATP5MC1* (UniProt accession P05496), *ATP5MC2* (UniProt accession Q06055), and *ATP5MC3* (UniProt accession P48201), respectively. These proteins are c-subunit isoforms that assemble into the c-ring in the F_0_ rotor. Subunit c is encoded by these three genes, though they only differ in their cleavable mitochondrial targeting peptides, resulting in generally identical mature proteins [12,22]. These proteolipid subunits are the most hydrophobic among the F_0_ subunits in this study, together forming the rotating ring that converts proton movement into mechanical torque. The mechanism works as conserved acidic residues in the c-subunits bind and release protons during catalysis, resulting in a stepwise rotation relative to ATP6 that drives conformational changes through the central stalk into the F1 sector [3,4,12]. Recent studies have connected mutations to multiple conditions, including cardiomyopathy and autosomal dominant spastic paraplegia and dystonia [23,24].

These eight subunits span distinct structural and functional categories within the ATP synthase F0 membrane sector. ATP6 is a core proton-translocating component, forming the stator interface adjacent to the rotating c-ring and contributing to the protein half-channels. AT5G1, AT5G2, and AT5G3 encode c-subunit isoforms that build up the c-ring. ATP8 stabilizes the membrane domain as an accessory membrane subunit. ATP68, ATPK, and ATPMK are smaller supernumerary and peripheral subunits that contribute to complex assembly, dimer stability, and membrane-domain architecture rather than catalyzing proton translocation [3,5,8–10].

The proteins were selected as a limited set because they are strongly membrane-associated, contain defined transmembrane regions suitable for QTY substitution, and could be evaluated using either complete CryoEM-derived native structures or AlphaFold3-predicted reference native models. The study aims to examine diverse test cases rather than a comprehensive analysis of all F_0_-associated subunits. Other key membrane-associated proteins, such as subunit b and additional peripheral-stalk components, were not chosen because the study prioritized a manageable subset of proteins. The exclusion potentially limits the ability of these results to be generalized to the complete ATP synthase membrane complex.

ATP synthase F_0_ subunits are membrane-embedded and hydrophobic, making them challenging to study without the use of detergents. The QTY code addresses this problem by reducing hydrophobicity, potentially converting hydrophobic proteins into candidate water-soluble analogues while seeking to preserve overall alpha-helix structure [25–27]. In this approach, selected hydrophobic residues in transmembrane regions are substituted with structurally similar polar residues: leucine (L) is replaced by glutamine (Q), isoleucine (I) and valine (V) by threonine (T), and phenylalanine (F) by tyrosine (Y). These substitutions reduce the calculated hydrophobicity, maintain similar protein structure, and may facilitate future detergent-free studies if resulting proteins can be shown to be soluble and structurally stable [25–28].

Recent advancements in cryogenic electron microscopy (CryoEM) have enabled high-resolution visualization of native membrane protein assemblies, such as ATP synthase, that have been previously difficult to stabilize [29,30]. With these new developments, the molecular structure of human ATP synthase has been experimentally determined, split into three distinct states that represent different snapshots during its rotation, with roughly ~120° between each state: State 1 (Protein Data Bank [PDB]: 8H9S) [31], state 2 (PDB: 8H9T) [32], and state 3, split into state 3a (PDB: 8H9U) [33] and state 3b (PDB: 8H9V) [34–36]. For our study, we will be using state 1. However, these experimentally determined structures don’t contain all subunits, meaning we have to rely on computational tools like AlphaFold3 for full comparisons.

AlphaFold-based structure prediction has created opportunities to study membrane proteins in silico. After the release of AlphaFold2 in 2021, the QTY code has been applied to G protein-coupled receptors, glucose transporters, solute carrier transporters, ABC transporters, neurological transporters such as serotonin, dopamine, and norepinephrine transporters, and glutamate transporters [37–43]. Reverse-QTY designs have also been used on human serum albumin for antitumor drug release and on the water-soluble CXCR4QTY receptor in a biomimetic sensing platform [44,45]. AlphaFold3, released in 2024, extends structure predictions beyond single proteins and is now capable of predicting complex biomolecular interactions and behavior [46]. Using AlphaFold3, the QTY code has also been applied to six human integral membrane enzymes and modeled ligand interactions [28].

Here, we present bioinformatic studies of four experimentally determined and four AlphaFold3-predicted human ATP synthase F_0_ subunits and their AlphaFold3-predicted QTY variants with reduced hydrophobicity. We provide superpositions of the hydrophobic native subunits and their reduced-hydrophobicity QTY variants. We also provide the comparative hydrophobicity of molecular structures with their hydrophilic QTY variants.

Despite major advancements in structural biology, membrane proteins remain challenging to characterize because their hydrophobic regions often require detergents for extraction, stabilization, and structural analysis [47]. This challenge is particularly present in ATP synthase F_0_, since it contains multiple membrane-embedded subunits, where precise positioning is required for proton translocation, oligomerization, and complex assembly. Previous studies have shown that the use of detergents can influence ATP synthase activity and proton pathway integrity, and isolated F_0_ subunits like the c-ring require special strategies for experimental characterization [48,49]. Therefore, approaches like the QTY code that aims to reduce hydrophobicity while retaining core structure may provide complementary strategies for studying membrane-embedded bioenergetic complexes.

Unlike previous QTY studies that have targeted individual protein receptors, transporters, and membrane enzymes, this study targets selected components of human ATP Synthase F_0_, a multisubunit rotary bioenergetic complex where function relies on proper subunit coordination and interactions. The eight chosen proteins represent distinct structural and functional contexts within the system, including components of the proton-translocating core, c-ring, accessory membrane domain, and supernumerary subunit architecture. By extending QTY-based design to ATP synthase, this study examines whether hydrophobicity reduction can be achieved while maintaining predicted structural similarity across a more diverse membrane protein context. These results provide some of the computational foundation for future experiments investigating the purification, expression, assembly, and biochemical characterization of QTY-designed ATP synthase F_0_ analogues.

## Results and discussion

### Rationale of the QTY Code on transmembrane helices

Integral transmembrane enzymes are notoriously difficult to study in aqueous solution because their transmembrane helices consist of hydrophobic residues essential for membrane localization. The amino acids Q and L, T and V/I, and Y and F share similar electron density maps [25,26]. The QTY code utilizes this similarly, and systematic replacements are made to reduce the hydrophobicity of integral transmembrane enzymes. The QTY code replaces hydrophobic amino acids with hydrophilic ones: leucine (L) with glutamine (Q), isoleucine (I) and valine (V) with threonine (T), and phenylalanine (F) with tyrosine (Y). Through these substitutions, the hydrophobic surface of the transmembrane regions is reduced without introducing major changes in residue size or helical packing tendency. Although sequence changes are introduced, QTY analogues remain similar to their native counterparts in isoelectric point (pI) and molecular weight (MW) (Fig 1).

**Fig 1:**
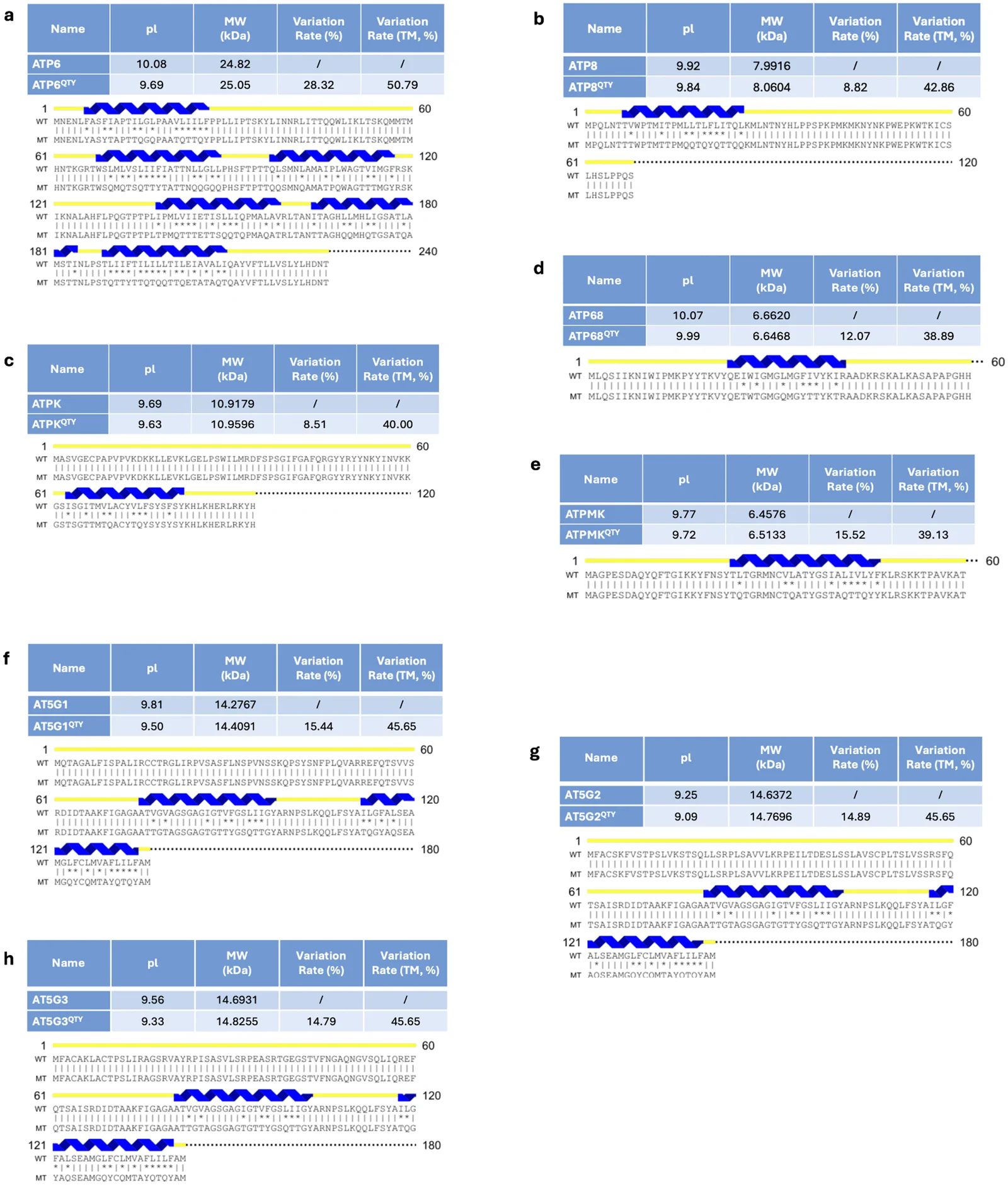
Protein sequence alignments of eight native ATP synthase F_0_ subunits with their QTY analogues. The symbols | and * indicate whether amino acids are identical or different, respectively. Please note the Q, T, and Y amino acids replacing L, V and I, and F, respectively. The alpha helices (blue) are shown above the protein sequences. The characteristics of native and QTY variants listed are isoelectric focusing point (pI), molecular weight (MW), total variation %, and transmembrane variation %. The alignments are: (A) ATP6 vs ATP6^QTY^, (B) ATP8 vs ATP8^QTY^, (C) ATPK vs ATPK^QTY^ (D) ATP68 vs ATP68^QTY^, (E) ATPMK vs ATPMK^QTY^, (F) AT5G1 vs AT5G1^QTY^, (G) AT5G2 vs AT5G2^QTY^, and (H) AT5G3 vs AT5G3^QTY^. Although there are substantial QTY changes in the transmembrane alpha helices (38.89–50.79%), the changes in MW and pI remain small. Protein names correspond to abbreviated manuscript labels defined in Table 1.

### Protein sequence alignments and other characteristics

The amino acid sequences of ATP6, ATP8, ATPK, ATP68, ATPMK, AT5G1, AT5G2, and AT5G3 were aligned with their QTY analogues (Fig 1). Residues altered by the QTY code are marked by the “*” symbol. Overall, these substitutions led to amino acid changes ranging from 8.51% to 28.32% while the variation within the transmembrane domains ranged from 38.89% to 50.79% (Fig 1). Nevertheless, the pI values remained largely unchanged, since Q, T, and Y are neutral amino acids that do not introduce additional acidic or basic side chains. The MW values increased only slightly, which can be explained by the mass differences among the substituted residues: leucine (L, 131.17 Daltons) is lighter than glutamine (Q, 146.14 Daltons); isoleucine (I, 131.17 Daltons) is heavier whereas valine (V, 117.15 Daltons) is lighter than threonine (T, 119.12 Daltons); and phenylalanine (F, 165.19 Daltons) is lighter than tyrosine (Y, 181.19 Daltons). Additionally, the QTY substitutions replace carbon-rich hydrophobic side chains (Carbon, 12 Daltons) with oxygen- and nitrogen-bearing groups (-NH2, 14 Daltons and -OH, 16 Daltons), resulting in slight MW increases.

### AlphaFold3 predictions and model confidence

Structures of the QTY analogues and subunits without CryoEM structures were predicted using AlphaFold3 through the online AlphaFold server [46,50]. Model quality was assessed using the confidence metrics, including Local Distance Difference Test (pLDDT), predicted Template Modeling score (pTM), and Predicted Aligned Error (PAE). Because AlphaFold3-native models were used as the reference native structures for AT5G1, AT5G2, AT5G3, and ATPMK, their confidence metrics are presented separately in Fig 2.

**Fig 2:**
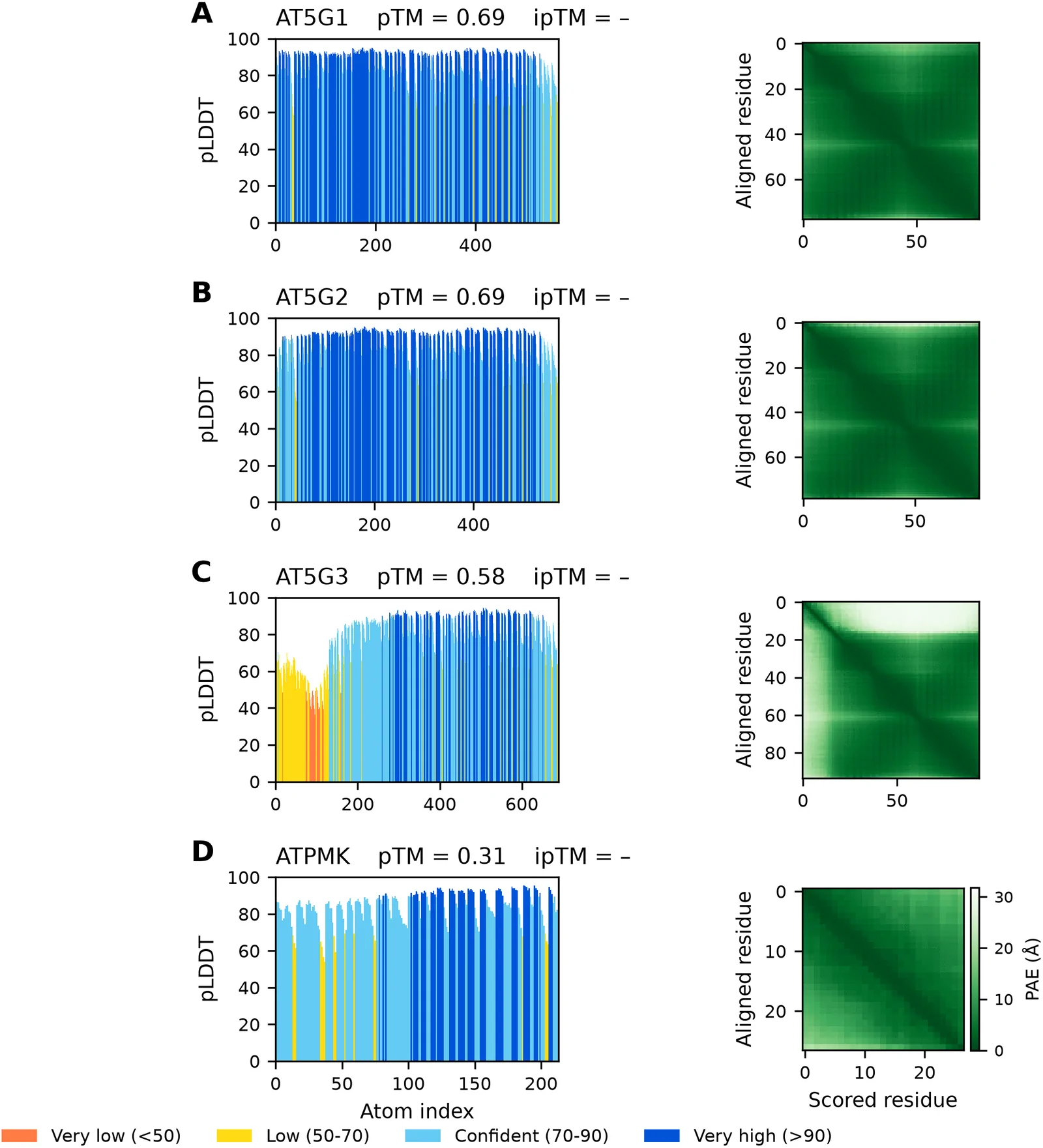
AlphaFold3 confidence summaries for ATP synthase F_0_ subunits without CryoEM structures. AlphaFold3-predicted native structures for (A) AT5G1^AF3^, (B) AT5G2^AF3^, (C) AT5G3^AF3^, and (D) ATPMK^AF3^ are shown with per-residue confidence (pLDDT), predicted aligned error (PAE) heatmaps, and predicted template modeling (pTM) scores. Confidence metrics were replotted from AlphaFold3 outputs using Python and Matplotlib. These AlphaFold3-predicted native structures were used as reference native models for native vs QTY structural and hydrophobicity surface comparisons in subunits without CryoEM structures. Unstructured loops were truncated.

The pLDDT measures the model’s confidence in each residue [46,51]. In AlphaFold3 predictions, dark blue indicates high confidence (pLDDT > 90), light blue indicates good confidence (pLDDT between 70 and 90), yellow indicates moderate confidence (pLDDT between 50 and 70), and orange indicates low confidence (pLDDT < 50) [46]. In our predictions, most transmembrane regions were dark or light blue, indicating greater model confidence in the helical regions, whereas terminal regions more often appeared yellow or orange, indicating greater flexibility or disorder (Fig 2) [52]. Whenever AlphaFold3 was used in a structural comparison, we excluded low-confidence and unstructured regions from both the predicted structure and the corresponding structure before analysis, so that the comparison focused on well-resolved and meaningful differences. The pTM score provides overall model confidence and expected global structural similarity [52]. In our study, across all predicted QTY analogues, the average pTM score was 0.732, indicating generally favorable overall AlphaFold3 confidence. Fig 2 specifically shows the AlphaFold3-native models used for the four subunits without experimental structures: AT5G1, AT5G2, AT5G3, and ATPMK, which had pTM scores of 0.69, 0.69, 0.58, and 0.31, respectively (Fig 2). PAE evaluates confidence in the relative positioning of residue pairs within the structure model [53]. In our predictions, the PAE plots were largely dominated by low-error regions, indicating high confidence in the relative spatial arrangement of most residues (Fig 2). Together, the pLDDT, pTM, and PAE results generally indicate favorable AlphaFold3 confidence metrics. Nevertheless, these models remain computational predictions and will require experimental validation to confirm their structures.

### Reference native structure selection

Not all subunits had complete experimental native CryoEM structures. Thus, ATP6, ATP8, ATPK, and ATP68 were compared using native CryoEM structures, while AT5G1, AT5G2, AT5G3, and ATPMK were compared using AlphaFold3-predicted native models. This ensured more consistent full-length protein comparisons between native and QTY structures. Further details are included in the Methods section.

### Superpositions of select CryoEM-determined native ATP synthase F_0_ subunits and their QTY analogues

We ask whether the molecular structure of four ATP synthase F_0_ transmembrane subunits can be superposed with their respective QTY analogues (Fig 3). The native structures of ATP6, ATP8, ATPK, and ATP68 (PDB: 8H9S) have been experimentally determined via CryoEM, whereas the QTY analogues were predicted via AlphaFold3. The superpositions are: ATP6^CryoEM^ *vs* ATP6^QTY_AF3^, ATP8^CryoEM^ *vs* ATP8^QTY_AF3^, ATPK^CryoEM^ *vs* ATPK^QTY_AF3^, and ATP68^CryoEM^ *vs* ATP68^QTY_AF3^.

**Fig 3:**
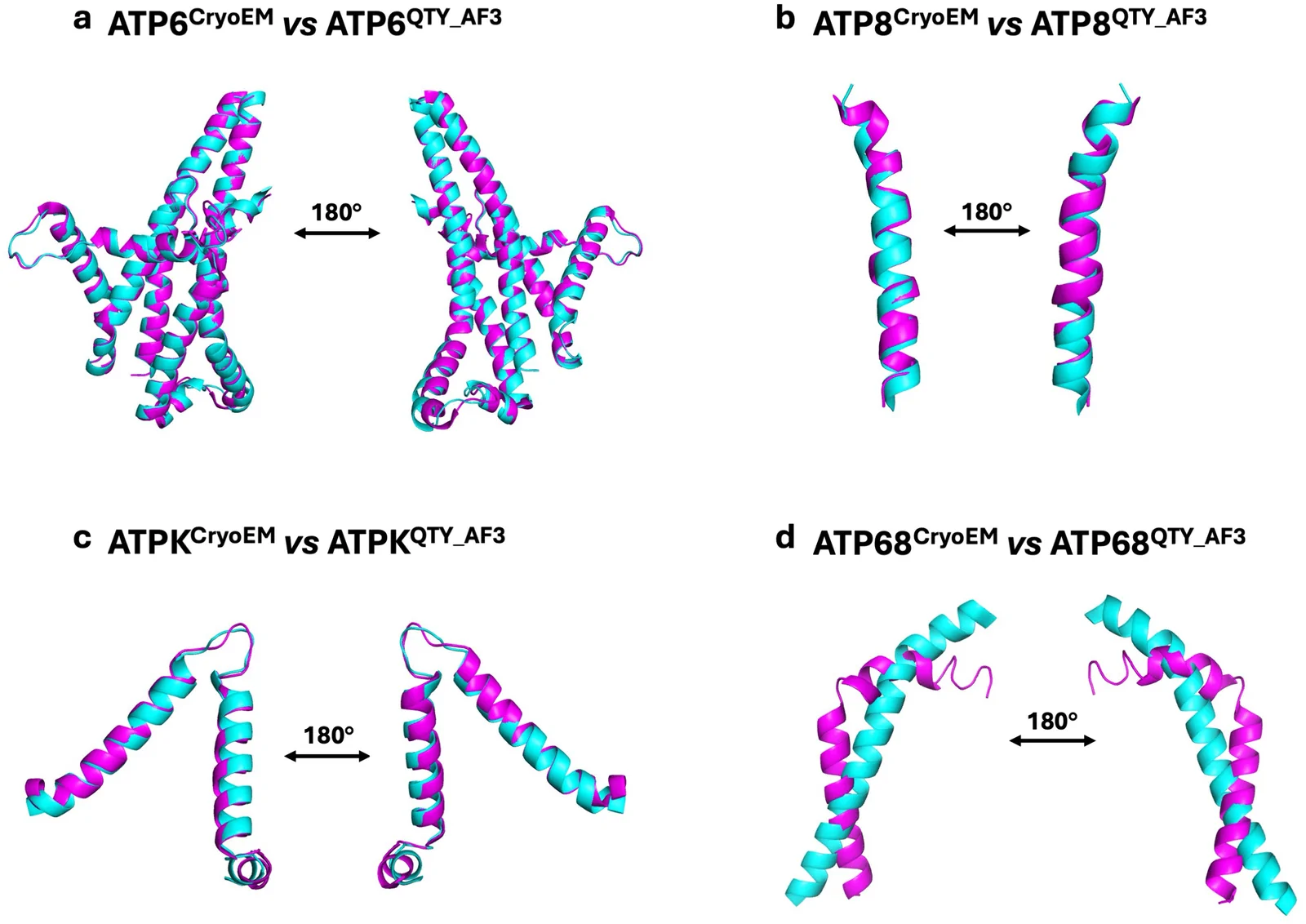
Superpositions of select CryoEM-determined native ATP synthase F_0_ subunits and their QTY analogues. The CryoEM-determined structures of the native subunits are obtained from the Protein Data Bank (PDB). CryoEM structures (magenta) are superposed with their QTY variants (cyan) predicted by AlphaFold3. Superpositions of ATP6, ATP8, and ATPK show that these native subunits share similar structure to their QTY variants. Unstructured loops with low confidence were removed. For ATP68, CryoEM structure was superposed with the unsegmented AlphaFold3-predicted QTY analog. The larger deviation reflects a bend in the CryoEM structure that is not present in the predicted model. Two views are shown for each protein (180°). Panels show: (A) ATP6^CryoEM^ vs ATP6^QTY-AF3^ (RMSD = 0.565 Å), (B) ATP8^CryoEM^ vs ATP8^QTY-AF3^ (RMSD = 2.546 Å), (C) ATPK^CryoEM^ vs ATPK^QTY-AF3^ (RMSD = 1.180 Å), and (D) ATP68^CryoEM^ vs ATP68^QTY-AF3^ (RMSD = 6.952 Å).

The native CryoEM structures and their AlphaFold3-predicted QTY analogues showed varying degrees of structural agreement. ATP6, ATP8, and ATPK yielded root-mean-square deviation (RMSD) values ranging from 0.565Å to 2.546Å (Fig 3, Table 2), specifically: a) ATP6^CryoEM^ *vs* ATP6^QTY-AF3^ (RMSD = 0.565Å), b) ATP8^CryoEM^ *vs* ATP8^QTY-AF3^ (RMSD = 2.546Å), and c) ATPK^CryoEM^ *vs* ATPK^QTY-AF3^ (RMSD = 1.180Å). As seen in Fig 3, these structures share similar overall folds despite a 40.00%–50.79% of replacements in transmembrane region amino acids using the QTY code.

**Table 2:** Protein characteristics of eight human ATP synthase F_0_ subunits and their QTY variants.

Name	Native–QTY RMSD (Å)	pI	MW (kDa)	TM (%)	Overall (%)
ATP6^CryoEM^	–	10.08	24.82	–	–
ATP6^QTY-AF3^	0.565	9.69	25.05	50.79	28.32
ATP8^CryoEM^	–	9.92	7.99	–	–
ATP8^QTY-AF3^	2.546	9.84	8.04	42.86	8.82
ATPK^CryoEM^	–	9.69	10.92	–	–
ATPK^QTY-AF3^	1.180	9.63	10.96	40.00	8.51
ATP68^CryoEM^	–	10.07	6.66	–	–
ATP68^QTY-AF3^	6.952	9.99	6.65	38.89	12.07
ATP68 residues 2–20	1.608	–	–	–	–
ATP68 residues 21–42	0.790	–	–	–	–
ATPMK^AF3^	–	9.77	6.46	–	–
ATPMK^QTY-AF3^	0.204	9.72	6.51	39.13	15.52
AT5G1^AF3^	–	9.81	14.28	–	–
AT5G1^QTY-AF3^	0.252	9.50	14.41	45.65	15.44
AT5G2^AF3^	–	9.25	14.64	–	–
AT5G2^QTY-AF3^	0.271	9.09	14.77	45.65	14.89
AT5G3^AF3^	–	9.56	14.69	–	–
AT5G3^QTY-AF3^	0.297	9.33	14.82	45.65	14.79

Abbreviations: pI, isoelectric point; MW, molecular weight; TM, transmembrane; AF3, AlphaFold3; CryoEM, cryo-electron microscopy; –, not applicable; RMSD, root-mean-square deviation. The eight ATP synthase F_0_ subunits are listed in the same order as Fig 1. Native structures for ATP6, ATP8, ATPK, and ATP68 were derived from the CryoEM human ATP synthase structure (PDB: 8H9S), whereas native structures for ATPMK, AT5G1, AT5G2, and AT5G3 were predicted by AlphaFold3. RMSD values are reported on the QTY row for each pair of native vs QTY comparisons. RMSDs were calculated after truncating unresolved and low-confidence regions in native proteins and corresponding residues in QTY analogues. For ATP68, the unsegmented RMSD of 6.952Å is reported as the primary comparison. Secondary segment-based superpositions of residues 2–20 and 21–42 yielded RMSDs of 1.608Å and 0.790Å, respectively. QTY substitutions within TM regions are substantial, ranging from 38.89% to 50.79%, while overall sequence changes range from 8.51% to 28.32%.

For ATP68, the CryoEM and unsegmented AlphaFold3-predicted QTY analog yielded a substantially higher RMSD of 6.952Å. This difference is primarily due to a bend in the CryoEM structure that isn’t captured in the isolated AlphaFold3-predicted model. When the two helical regions were superposed separately, residues 2–20 and 21–42 yielded RMSDs of 1.608Å and 0.790 Å, respectively, indicating better local alignment (S1A Fig). The unsegmented RMSD is reported as the primary comparison, while the segment-based values support local structural similarity.

Importantly, the CryoEM-native vs AlphaFold3-native ATP68 comparison also showed a high RMSD of 6.443Å, whereas the AlphaFold3-native vs AlphaFold3-QTY comparison yielded an RMSD of 1.907Å. Therefore, the major conformational difference is already present between the experimental CryoEM structure and the isolated AlphaFold3-native prediction and cannot be attributed primarily to the QTY substitutions.

The origin of the bend in the CryoEM model and lack thereof in the AlphaFold3 model cannot be fully established from the present analysis. In the assembled CryoEM complex, ATP68 is closely associated primarily with neighboring ATP8 and ATP6 (S2A Fig). Notably, ATP68 Tyr21, located at the bend junction, lies approximately 2.9Å from the carbonyl oxygen of ATP8 Gln25, consistent with a possible hydrogen bond (S2B Fig). Such inter-subunit contacts could help constrain or stabilize the bent ATP68 conformation within the assembled ATP synthase complex. This is consistent with studies showing that subunit-subunit interactions within ATP synthase F_0_ sector can contribute to complex stability and that disruptions can destabilize the assembly [3]. The conformational difference may also reflect the specific membrane environment or rotational/conformational state captured experimentally, whereas the isolated ATP68 structure predicted in AlphaFold3 lacks such constraints.

To further examine whether QTY substitutions occur at functionally important interfaces, ATP6 substitutions were mapped onto the ATP6–c-ring interface in the assembled human ATP synthase F₀ complex. 14 QTY-substituted ATP6 residues were located within 5 Å of the c-ring (S3 Fig). ATP6 Arg159 and c-ring Glu58 form a functionally important electrostatic interaction during proton translocation, with Arg159 helping deprotonate Glu58 while also acting as an electrostatic barrier between the proton half-channels. These key residues were not altered by QTY substitutions [3,4,14]. In the native structure, ATP6 Arg159 displayed a 2.91 Å sidechain-to-sidechain distance to the nearest c-subunit Glu58 (S3B Fig). Arpeggio contact analysis was further conducted to examine the native ATP6-c-ring interactions involving the 14 QTY-code-targeted residues in close proximity of the c-ring (S3C Fig). ATP6 Arg159’s contact with Glu58 was classified as hydrogen-bonding, ionic, and polar. However, preservation of these residues does not establish the preservation of their interactions or proton-translocation function, because the QTY substitutions were not modeled in the context of a fully assembled QTY ATP6 and c-ring complex.

### Superpositions of select AlphaFold3-predicted ATP synthase F_0_ subunits with experimental data and their QTY analogues

Although these four ATP synthase F_0_ subunits have experimentally determined CryoEM native structures available, we performed an additional test by superposing their AlphaFold3-predicted native structures with their AlphaFold3-predicted QTY analogues (Fig 4). Both the native structure and QTY analogues were predicted via AlphaFold3. The structures superposed well with low RMSD, specifically: a) ATP6^AF3^ *vs* ATP6^QTY-AF3^ (RMSD = 0.341 Å), b) ATP8^AF3^ *vs* ATP8^QTY-AF3^ (RMSD = 0.238Å), c) ATPK^AF3^ *vs* ATPK^QTY-AF3^ (RMSD = 2.583 Å), and d) ATP68^AF3^ *vs* ATP68^QTY-AF3^ (RMSD = 1.907Å). For ATP68, unsegmented versions of both AlphaFold3-predicted models were used, and the AlphaFold3-native versus AlphaFold3-QTY comparison yielded a substantially lower RMSD than the CryoEM-native versus AlphaFold3-QTY comparison of 6.952 Å. As a secondary local analysis, the segmented AlphaFold3-native vs AlphaFold3-QTY comparison yielded RMSDs of 0.955Å and 0.384Å for residues 2–20 and 21–42, respectively (S1B Fig).

**Fig 4:**
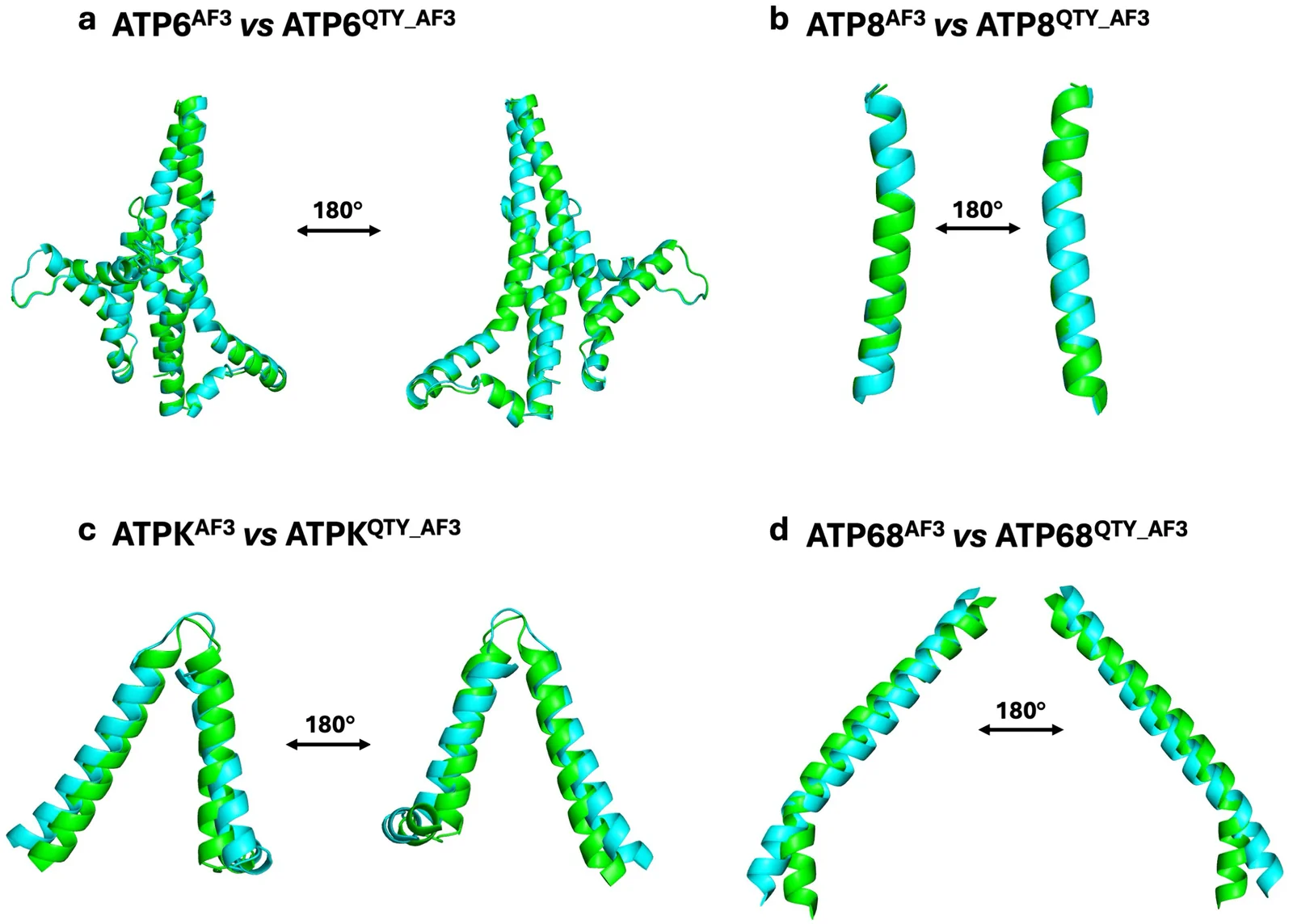
Superpositions of select AlphaFold3-Predicted native ATP synthase F_0_ subunits with experimental data and their QTY analogues. AlphaFold3-predicted native structures (green) are superposed with their corresponding QTY variants (cyan). Unstructured loops with low confidence were removed. Two views are shown for each protein (180°). Two views are shown for each protein (180°). Panels show: (A) ATP6^AF3^ vs ATP6^QTY-AF3^ (RMSD = 0.341 Å), (B) ATP8^AF3^ vs ATP8^QTY-AF3^ (RMSD = 0.238 Å), (C) ATPK^AF3^ vs ATPK^QTY-AF3^ (RMSD = 2.583 Å), and (D) ATP68^AF3^ vs ATP68^QTY-AF3^ (RMSD = 1.907 Å).

Because neither of the AlphaFold3 models reproduced the bend observed in the CryoEM structure, the high RMSD in the unsegmented comparison is due to the difference between the CryoEM model and AlphaFold3 predictions, not attributable solely to QTY substitutions. The segmented analysis shows high local structural similarity, consistent with previous comparisons. Since both structures were generated within the same AlphaFold3 framework, these comparisons show similar AlphaFold3 predictions for native and QTY sequences.

### Superpositions of select CryoEM-determined native ATP synthase F_0_ subunits with their AlphaFold3-predicted native structures and QTY analogues

For ATP synthase F_0_ subunits with available CryoEM structures, we superposed i) all available CryoEM-determined native ATP synthase F_0_ subunits ii) AlphaFold3-predicted native ATP synthase F_0_ subunits and iii) AlphaFold3-predicted QTY analogues. For ATP6, ATP8, and ATPK, the three different structures superposed well (Fig 5). These superpositions indicate modelled similarity between experimentally determined structures and their QTY analogues, and between AlphaFold3-predicted native structures and QTY analogues (Fig 1 and Fig 3). Direct CryoEM-native vs AlphaFold3-native superpositions were analyzed too, yielding reasonable RMSDs of 0.500 Å for ATP6, 2.882Å for ATP8, and 2.886Å for ATPK.

**Fig 5:**
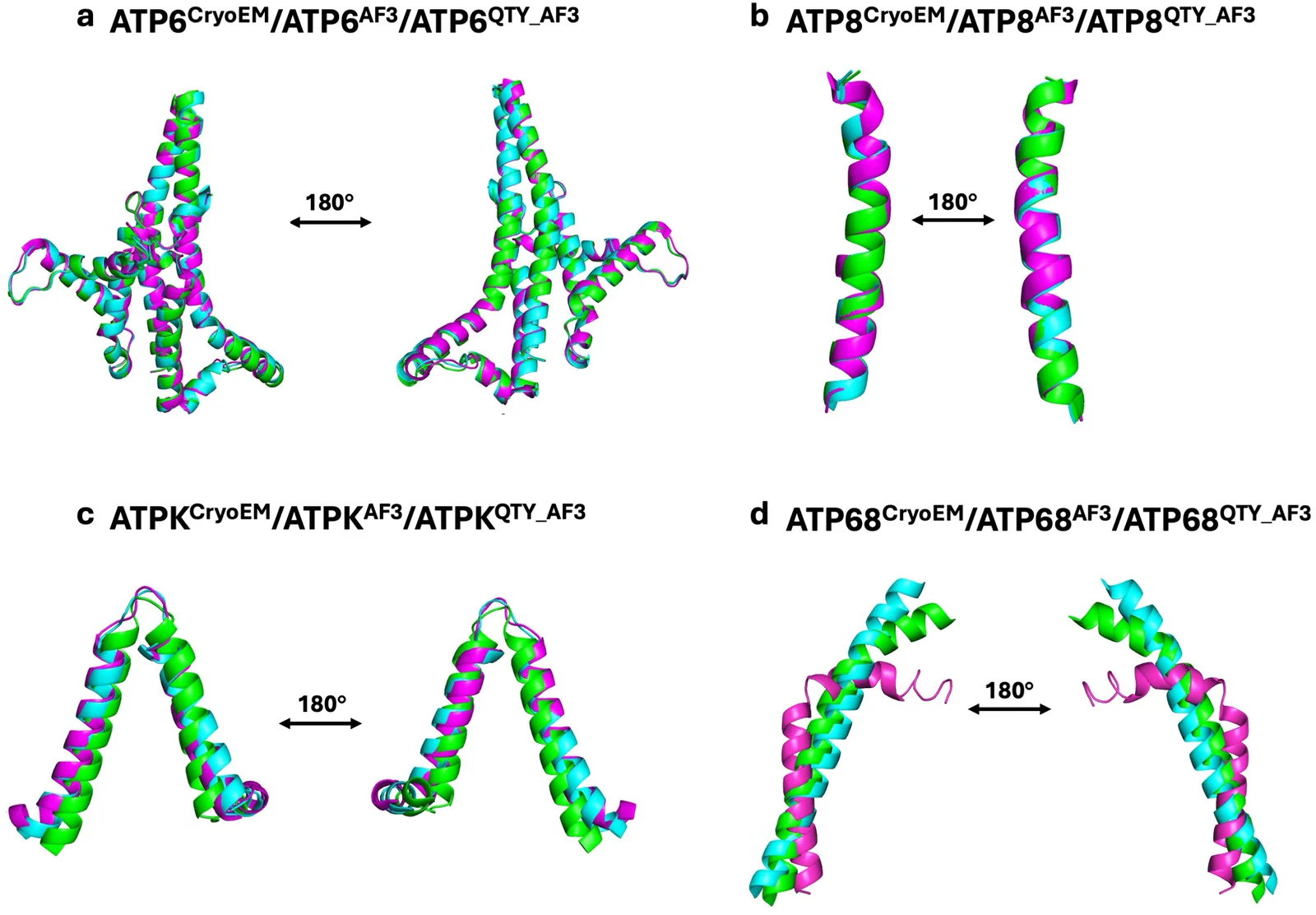
Superpositions of select CryoEM-determined native ATP synthase F_0_ subunits with their AlphaFold3-predicted native structures and QTY analogues. Superposition of i) all available CryoEM-determined native ATP synthase F_0_ subunits (magenta) with ii) select AlphaFold3-predicted native ATP synthase F_0_ subunits (green) and iii) select AlphaFold3-predicted water-soluble QTY analogs (cyan). Individual superpositions are shown in Figs 4 and 3. For ATP6, ATP8, and ATPK, all three structures superposed well visually, with insignificant differences shown. ATP68 shows pronounced differences between the CryoEM structure and both AlphaFold3 models. Two views are shown for each protein (180°). Panels show: (A) ATP6^CryoEM^/ATP6^AF3^/ATP6^QTY-AF3^, (B) ATP8^CryoEM^/ATP8^AF3^/ATP8^QTY-AF3^, (C) ATPK^CryoEM^/ATPK^AF3^/ATPK^QTY-AF3^, and (D) ATP68^CryoEM^/ATP68^AF3^/ATP68^QTY-AF3^. Direct CryoEM-native vs AlphaFold3-predicted native RMSDs were also calculated for ATP6^CryoEM^ vs ATP6^AF3^ (0.500Å), ATP8^CryoEM^ vs ATP8^AF3^ (2.882Å), ATPK^CryoEM^ vs ATPK^AF3^ (2.886Å), and ATP68^CryoEM^ vs ATP68^AF3^ (6.443Å).

Meanwhile, ATP68 demonstrated less structural similarity, with a direct CryoEM-native vs AlphaFold3-native superposition yielding an RMSD of 6.443 Å. Consistent with previously established results, the CryoEM model of ATP68 displayed the bend, whereas the AlphaFold3-native and AlphaFold3-QTY models did not, thus leading to greater structural deviation.

### Superpositions of select AlphaFold3-predicted native ATP synthase F_0_ subunits without experimental data and their QTY analogues

Since some subunits lacked CryoEM structures, and the preceding comparisons showed selected AlphaFold3-predicted native models reproduce the general backbone structure of corresponding CryoEM structures, we ask whether the AlphaFold3-predicted molecular structure of four ATP synthase F_0_ transmembrane subunits can be superposed with their respective QTY analogues (Fig 6). The native structures that lacked experimental data of AT5G1, AT5G2, AT5G3, and ATPMK were predicted via AlphaFold3, and their QTY analogues were also predicted via AlphaFold3. The superpositions are: AT5G1^AF3^ *vs* AT5G1^QTY-AF3^, AT5G2^AF3^ *vs* AT5G2^QTY-AF3^, AT5G3^AF3^ *vs* AT5G3^QTY-AF3^, and ATPMK^AF3^ *vs* ATPMK^QTY-AF3^.

**Fig 6:**
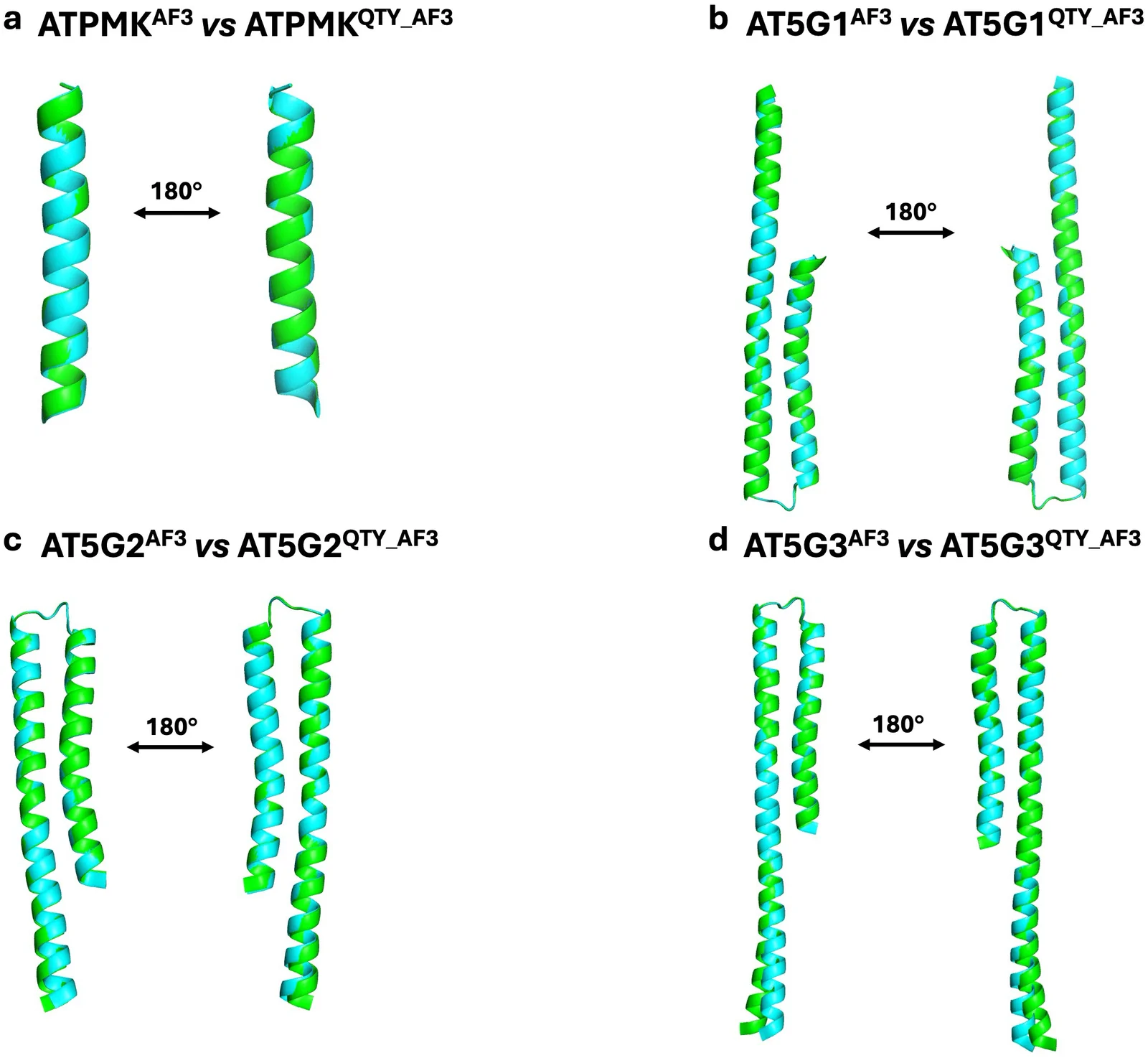
Superpositions of select AlphaFold3-predicted native ATP synthase F_0_ subunits without experimental data and their QTY analogues. AlphaFold3-predicted native structures (green) are superposed with their corresponding QTY variants (cyan). Unstructured loops with low confidence were removed. Two views are shown for each protein (180°). Panels show: (A) ATPMK^AF3^ vs ATPMK^QTY_AF3^ (RMSD = 0.204 Å), (B) AT5G1^AF3^ vs AT5G1^QTY-AF3^ (RMSD = 0.252 Å), (C) AT5G2^AF3^ vs AT5G2^QTY-AF3^ (RMSD = 0.271 Å), and (D) AT5G3^AF3^ vs AT5G3^QTY-AF3^ (RMSD = 0.297 Å). Note that structural comparisons between AlphaFold3 models demonstrate consistency between predicted models rather than independent structural validation.

The AlphaFold3-predicted native structures and their AlphaFold3-predicted QTY analogues superposed well. The RMSD values ranged from 0.204Å to 0.297Å (Fig 6, Table 2), specifically: a) AT5G1^AF3^ *vs* AT5G1^QTY-AF3^ (RMSD = 0.252Å), b) AT5G2^AF3^ *vs* AT5G2^QTY-AF3^ (RMSD = 0.271Å), c) AT5G3^AF3^ *vs* AT5G3^QTY-AF3^ (RMSD = 0.297Å), and d) ATPMK^AF3^ *vs* ATPMK^QTY-AF3^ (RMSD = 0.204Å). Because both structures in the comparison were generated via AlphaFold3, these results should be interpreted as consistency within the same prediction framework rather than independent structural validation.

### Additional structural and membrane-placement analyses

To assess structural agreement beyond RMSD, native and QTY structures were further compared using TM-score, aligned-residue counts, α-helical content, membrane-placement parameters including hydrophobic thickness, tilt, and ΔG_transfer_ (S1 Table). Several subunits showed high global structural agreement, including ATP6, AT5G1, AT5G2, and AT5G3, with TM-scores of 0.998, 0.989, 0.992, and 0.990, respectively. These subunits also displayed near complete alignment. Across all subunits, QTY analogues generally maintained high α-helical content, demonstrating the conservation of helical architecture despite transmembrane substitutions. ATP68 showed lower global structural agreement, which can be explained by the conformation difference discussed above. Its local helical segments maintained substantial α-helical character and showed lower RMSDs, further supporting strong local agreement and preservation of secondary structure despite global differences.

PPM 3.0 analysis showed reductions in predicted hydrophobic thickness and less negative membrane-transfer free energies, ΔG_transfer_, for the QTY analogues (S1 Table). Since the QTY code is designed to reduce membrane affinity while preserving overall fold, these changes are consistent with the intended reduction in hydrophobicity and membrane affinity produced by the QTY substitutions. Changes in predicted membrane tilt were also observed, reflecting the altered physicochemical properties of the QTY analogues.

Since AT5G1, AT5G2, AT5G3, and ATPMK do not have experimental CryoEM structures, AlphaFold3-native models were used in structural comparisons. Stereochemical analysis was performed on these AlphaFold3-native models using MolProbity (S2 Table). AT5G1, AT5G2, and AT5G3 had MolProbity scores of 0.50, 1.13, and 1.21, respectively, 100% of residues in favored Ramachandran regions, and no Ramachandran outliers. AT5G1 and AT5G3 also showed no rotamer outliers, while AT5G2 had 1.82% rotamer outliers. ATPMK showed relatively weaker stereochemical metrics, with a MolProbity score of 2.95, 91.67% Ramachandran-favored residues, 4.17% Ramachandran outliers, and 18.18% rotamer outliers. However, due to the short sequence of ATPMK after truncation, even a small number of unfavorable residues could result in relatively large percentages, so the ATPMK model was interpreted with more caution. MolProbity provides us with additional assessment of model geometry but does not independently validate the structural accuracy of AlphaFold3 predictions.

### Analysis of the hydrophobic surface of CryoEM-determined and AlphaFold3-predicted native ATP synthase F_0_ subunits and their QTY analogues

The eight ATP synthase F_0_ transmembrane subunits selected are inherently hydrophobic and water-insoluble, especially in the transmembrane alpha helical domains. The eight proteins have an average hydrophobicity of 0.2959 in their transmembrane regions. Typically, for protein studies, detergents are used to solubilize the native proteins after removing them from the lipid bilayer. Without proper tools, these proteins can aggregate, precipitate, or denature in aqueous solutions.

Hydrophobic surface areas are highlighted in yellow (Fig 7, Fig 8), embedded in the hydrophobic lipid bilayer. Here, Leucine (L), Isoleucine (I), Valine (V), and Phenylalanine (F) interact with lipid molecules to exclude water, leading to a hydrophobic and nonpolar patch.

**Fig 7:**
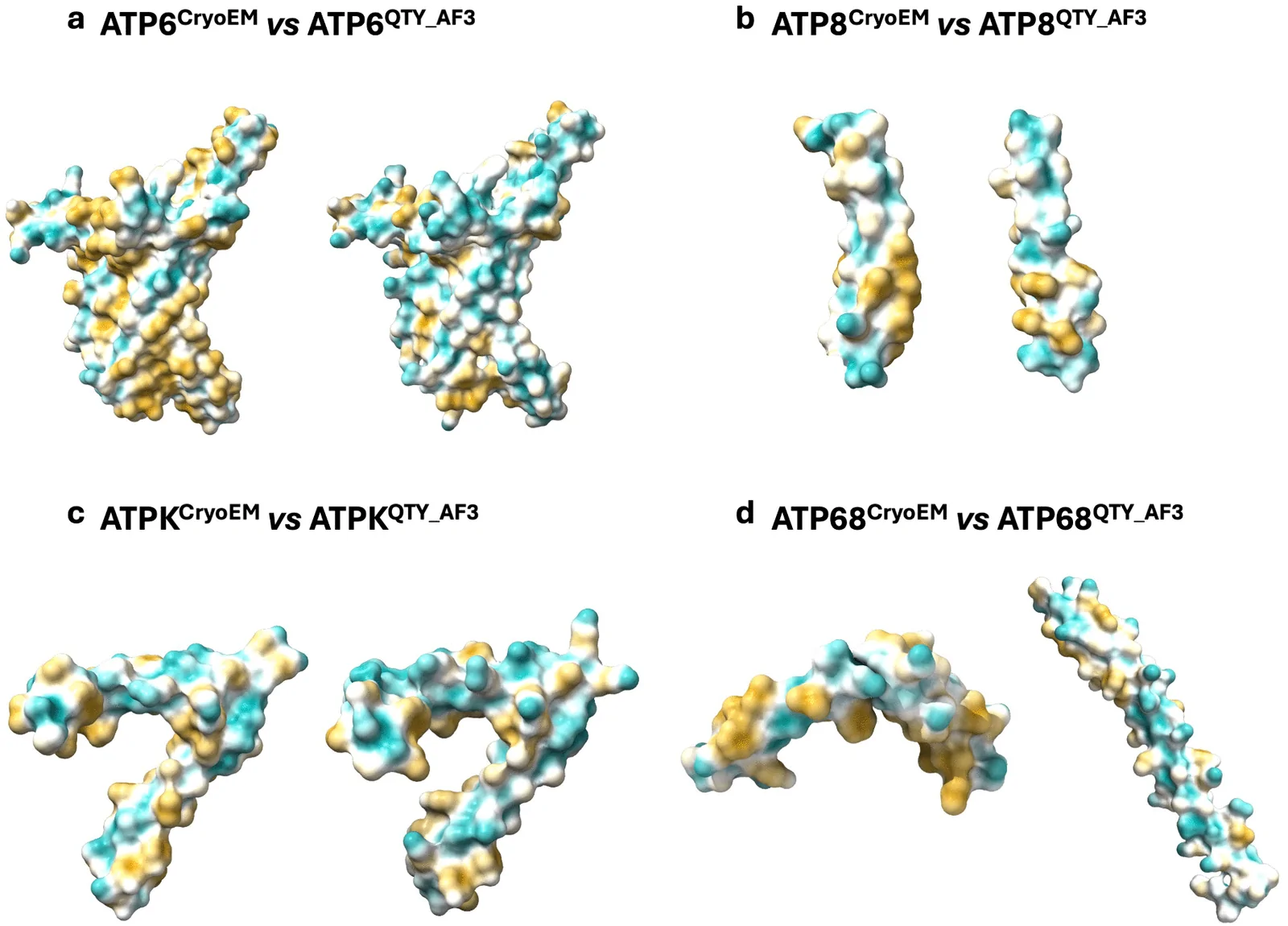
Hydrophobic surface comparison of CryoEM-determined ATP synthase F_0_ subunits and AlphaFold3-predicted QTY analogues. Molecular surface hydrophobicity is mapped as hydrophobic (brown) and hydrophilic (cyan). The native subunits have many hydrophobic residues L, I, V, and F in their transmembrane helices. QTY variants were generated by substituting L → Q, I/V → T, and F → Y to reduce transmembrane hydrophobicity. Unstructured loops with low confidence were removed. Two views are shown for each protein (180°). Panels show: (A) ATP6^CryoEM^ vs ATP6^QTY-AF3^, (B) ATP8^CryoEM^ vs ATP8^QTY-AF3^, (C) ATPK^CryoEM^ vs ATPK^QTY-AF3^, and (D) ATP68^CryoEM^ vs ATP68^QTY-AF3^.

**Fig 8:**
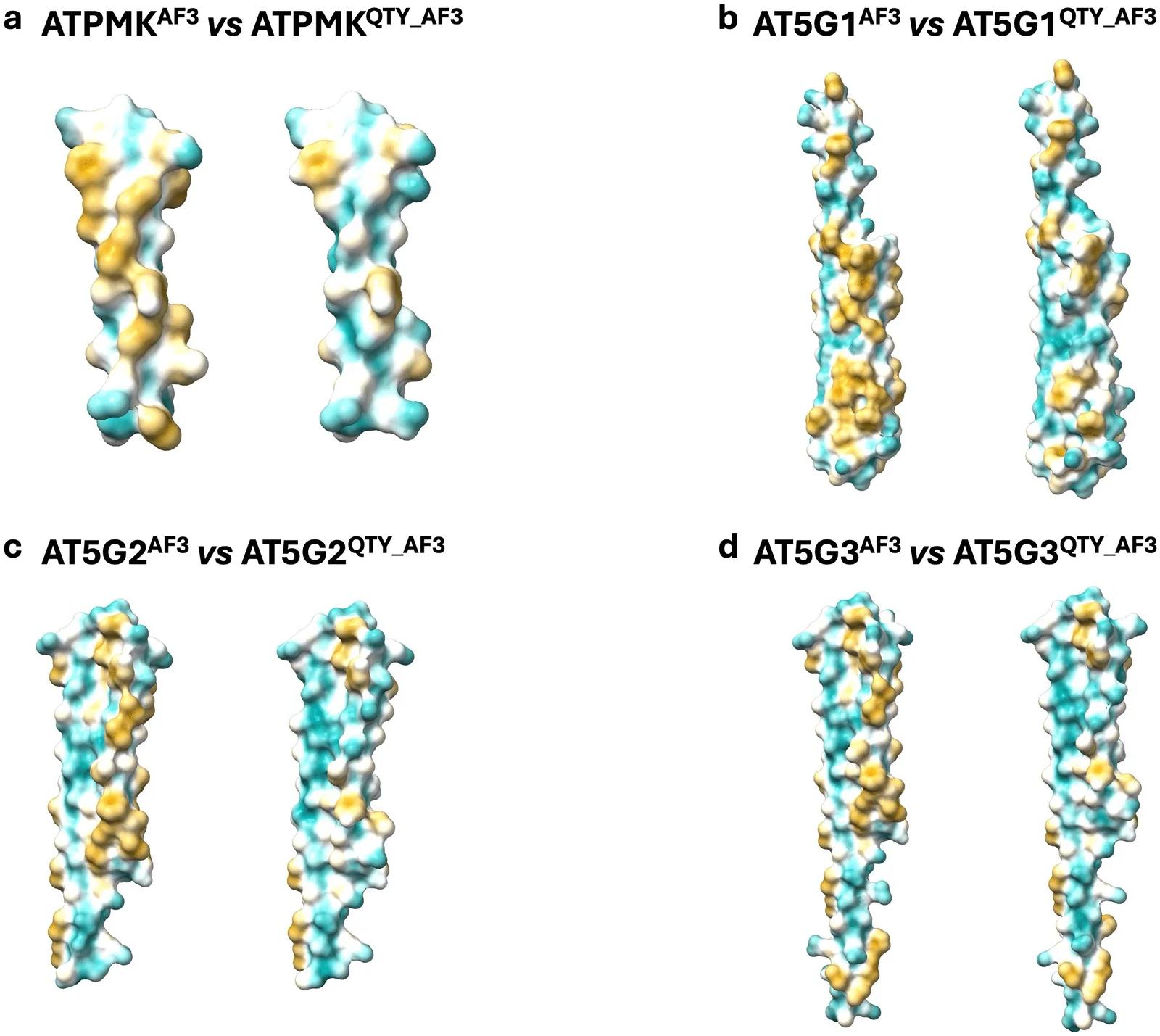
Hydrophobic surface comparison of AlphaFold3-predicted native ATP synthase F_0_ subunits and their QTY analogues. Molecular surface hydrophobicity is mapped as hydrophobic (brown) and hydrophilic (cyan). The native subunits have many hydrophobic residues L, I, V, and F in their transmembrane helices. QTY variants were generated by substituting L → Q, I/V → T, and F → Y to reduce transmembrane hydrophobicity. Unstructured loops with low confidence were removed. Two views are shown for each protein (180°). Panels show: (A) ATPMK^AF3^ vs ATPMK^QTY_AF3^, (B) AT5G1^AF3^ vs AT5G1^QTY-AF3^, (C) AT5G2^AF3^ vs AT5G2^QTY-AF3^, and (D) AT5G3^AF3^ vs AT5G3^QTY-AF3^.

By applying the QTY code, we systematically replaced hydrophobic amino acids L, I/V, and F, with hydrophilic amino acids Glutamine (Q), Threonine (T), and Tyrosine (Y), reducing hydrophobic surfaces. The average hydrophobicity of the eight proteins decreased from 0.2959 in their native variants to −1.1023 in the QTY analogues. The decrease in hydrophobicity was substantial, and the AlphaFold3 models of the QTY sequences retained a similar predicted overall alpha-helical structure. This is consistent with previous experiments, where QTY variants of chemokine and cytokine receptors were shown to retain structural integrity, stability, ligand-binding ability, and become water-soluble.

### Rationale for selecting ATP synthase F_0_ subunits for our study

The QTY code can be applied to a wide range of membrane enzymes, including initial studies focusing on seven chemokine receptors and four cytokine receptors, using biophysical, biochemical, and molecular approaches [26,27,43]. This study expands on the demonstrated success of the QTY code, selecting eight components of the human mitochondrial ATP synthase F_0_ sector. The eight protein subunits were selected as a limited, criterion-based, non-random set: ATP6, ATP8, ATPK, ATP68, ATPMK, AT5G1, AT5G2, and AT5G3. They were chosen for their defined transmembrane regions and availability of suitable CryoEM structures or AlphaFold3 reference models.

As mentioned in the Introduction, these proteins occupy distinct structural and functional contexts within the F0 sector and are not intended to represent the complete ATP synthase assembly. ATP synthase itself is a key component for oxidative phosphorylation, part of cellular respiration, and defects in its membrane sector have been associated with impaired mitochondrial energy metabolism, severe neuromuscular diseases like Parkinson’s, Alzheimer’s, and Amyotrophic Lateral Sclerosis, and other diseases like fragile X syndrome, the fatal neurodegenerative Batten disease, and cardiovascular complications [54–56]. Additionally, the strongly hydrophobic nature of transmembrane alpha helices in F_0_ subunits makes them hard to study experimentally in aqueous solutions without detergents. This combination of functional importance and structural architecture makes the ATP synthase F_0_ sector a suitable system for testing whether QTY substitutions can still reduce hydrophobicity while preserving overall fold and enabling informative structural comparisons across a new type of protein system.

## Summary

The ATP synthase F_0_ sector is an essential and meaningful system in testing broader applications of the QTY code. These subunits form a membrane-embedded proton-translocating system for ATP synthase, a highly conserved and essential component of mitochondrial ATP production as part of oxidative phosphorylation during cellular respiration. Defects have been associated with a wide spectrum of human disease, including but not limited to neurodegenerative, neuromuscular, metabolic, and cardiovascular disorders [54–56]. For this reason, the ability to develop more hydrophilic yet structurally similar F_0_ subunit analogues becomes a crucial step for future biochemical and structural characterization [26,27,37,43].

Beyond reducing calculated hydrophobicity, QTY-designed analogues may provide a potential approach for studying ATP synthase components that have been difficult to characterize experimentally. Native F_0_ subunits require membrane environments and detergent-based approaches for extraction and stabilization, which can influence protein stability and complicate biochemical analysis [47]. If experimentally validated, more hydrophilic QTY analogues have the potential to complement existing methods by allowing for more controlled studies of membrane proteins like ATP synthase F_0_. For example, smaller and less-characterized accessory subunits like ATP68, ATPK, and ATPMK could be initial candidates for expression and purification studies. Meanwhile, ATP6 and c-subunit isoforms like AT5G1, AT5G2, and AT5G3 will likely require multisubunit systems due to their roles in proton translocation and c-ring assembly. These possibilities remain hypothetical and require experimental validation.

Future studies could expand by testing whether ATP synthase F_0_ subunits’ QTY analogues preserve the capability to interact with other subunits and conduct proton-transfer-related features required for assembly and function. ATP synthase comprises the membrane-embedded F_0_ rotor and F_1_ catalyst in the matrix, both requiring intimate and proper protein binding to function. Thus, ATP synthase function, specifically the F_0_ rotor, depends on interactions between multiple subunits. For example, ATP8, included in this study, interacts with subunit b, which was not included. In the native enzyme, ATP8 stabilizes the association of ATP6 with the ATP synthase peripheral stalk through binding with subunit b. Meanwhile, proton translocation occurs at the ATP6–c-ring interface. ATP6 and the c-subunit homologs AT5G1, AT5G2, and AT5G3 were included in this study, but further studies should evaluate these subunits as assembled proton-conducting interfaces [8–10,12].

More generally, similarity of the predicted backbone does not demonstrate preservation of proton translocation, rotor interaction, assembly, stability, or correct side-chain packing. These functions depend on specific residues, subunit interfaces, electrostatics, membrane positioning, and conformational dynamics. QTY substitutions introduce neutral polar side chains and therefore do not directly replace the charged ATP6 Arg159 or c-ring Glu58 residues, but substitutions at surrounding residues could potentially impact local interface interactions. The present study identifies the preservation of relevant residues but does not establish preservation of ATP synthase function. Future work with AlphaFold3 could model multimeric F_0_ sector complexes to test whether native-like interactions remain after QTY substitution [46].

Because the study is purely computational, the current conclusions should be interpreted within the scope of in silico structural bioinformatic experiments. AlphaFold3 confidence metrics provide information on model confidence, but experimental validation ought to be performed to determine whether ATP Synthase F_0_ subunits’ QTY analogues can be expressed as functioning water-soluble proteins [46]. ATPMK and AT5G1-3 did not have established CryoEM structures, and their comparisons were performed between AlphaFold3 native and QTY predictions, meaning the low RMSD reflects agreement between shared model parameters and does not independently reflect structural preservation. ATP68 also showed a substantial difference between its CryoEM model and isolated AlphaFold3 prediction, indicating that local helical similarity does not indicate preservation of the full complex-associated conformation.

Even with these limitations, the study shows that QTY substitutions can substantially reduce calculated hydrophobicity across structurally and functionally distinct ATP synthase F_0_ subunits while preserving native-like predicted structure in most comparisons. By extending the QTY code beyond previously studied membrane enzymes to components of a multisubunit bioenergetic membrane protein, these findings establish a computational foundation for selecting and prioritizing ATP synthase F_0_ analogues for future expression, structural assembly, and functional characterization.

## Conclusion

Three chemically distinct yet structurally similar alpha-helices are present in nature [25]: hydrophilic alpha-helices, occurring in water-soluble proteins like hemoglobin, lysosomes, growth factors, cytokines, and antibodies; hydrophobic alpha-helices, occurring in integral membrane proteins like G-protein coupled receptors, transporters, ion channels, and photosynthetic electron transport complexes; and amphiphilic alpha-helices, containing both hydrophobic and hydrophilic sides. These helices remain highly similar in core geometry, despite substantial differences in amino acid makeup and physicochemical properties. This principle provides the molecular basis for the QTY code [26].

In our study, we applied the QTY code to eight ATP Synthase F_0_ subunits: ATP6, ATP8, ATPK, ATP68, ATPMK, AT5G1, AT5G2, and AT5G3. We generated corresponding QTY analogues, comparing them with CryoEM- or AlphaFold3-generated native structures for bioinformatics analysis. We employed numerous in silico computational tools to analyze the sequences and structural characteristics. Although QTY substitutions introduced changes in amino acid sequence and composition, the native and QTY proteins remained similar in key properties, including isoelectric point and molecular weight. The predicted QTY structures retained similar overall backbone folds to their native structures. Preservation of functional properties such as proton-translocation activity, assembly competence, and aqueous solubility are not established and should still be verified further. QTY variants also showed significantly reduced calculated hydrophobicity and modelled hydrophobic surfaces, supporting their selection as candidates for future water-solubility testing. Additional TM-score, secondary-structure, membrane-placement, and stereochemical analyses further supported preservation of the overall helical framework while showing reduced predicted membrane compatibility in the QTY analogues. These results are consistent with previous studies of the QTY code, which have focused on chemokine receptors, cytokine receptors, ABC transporters, rhomboid proteases, and other integral membrane proteins [26,27,37–43].

## Methods

### Protein sequence alignments and other characteristics

We obtained the native amino acid sequences for eight human ATP synthase F_0_ proteins using UniProt (https://www.uniprot.org/) with the accessions listed in Table 1: P00846, P03928, P56378, P56134, Q96IX5, P05496, Q06055, and P48201. These correspond to the manuscript-specific protein labels defined in Table 1: ATP6, ATP8, ATPK, ATP68, ATPMK, AT5G1, AT5G2, and AT5G3. UniProt transmembrane-region annotations were used for the transmembrane regions and visualized as topology diagrams with Protter (https://wlab.ethz.ch/protter/) (S4 Fig). Reduced-hydrophobicity QTY variants were generated by applying the QTY code to native FASTA sequences using the Protein Solubility Server (PSS) (https://pss.sjtu.edu.cn/) [57]. The server also produced several other protein characteristics (e.g. MWs, pI values, hydrophobicity, and overall variation), which we used for comparison across native and QTY variants. We also used its predictions of transmembrane helix topology to compare the topology of native ATP synthase F_0_ subunits and their corresponding QTY variants (S5 Fig).

### Structural models

When available, native structures were retrieved from experimentally determined CryoEM models available in the RCSB Protein Data Bank (RCSB PDB), specifically state 1 of the human ATP synthase CryoEM structure (PDB 8H9S) (https://www.rcsb.org/structure/8H9S/), which was chosen for its singular and well-defined conformational structure. For proteins without an experimentally determined PDB structure, native models were generated via AlphaFold3 servers (https://alphafoldserver.com/) from corresponding UniProt sequences. These include ATPMK, AT5G1, AT5G2, and AT5G3, where both the native and QTY structures were generated via AlphaFold3. Systematic evaluation of different independent AlphaFold3 parameters was not performed. QTY analogues were generated in the same way, using QTY-designed sequences as inputs for AlphaFold3 predictions. AlphaFold3 confidence metrics were extracted from the prediction outputs and replotted in Python using Matplotlib for visualization of pLDDT, PAE, and pTM metrics.

### Rationale for separating comparisons into native CryoEM and AlphaFold3-predicted native structures

We separated structural comparisons into native CryoEM and native AlphaFold3 categories because experimental CryoEM structures were not available for all ATP synthase F_0_ subunits. ATP6, ATP8, ATPK, and ATP68 each had PDB entries with CryoEM structures, allowing us to compare their native CryoEM structures with their QTY analogues.

Meanwhile, ATPMK, AT5G1, AT5G2, and AT5G3 were analyzed using AlphaFold3-predicted native structures. Although AT5G1 had a PDB entry, its CryoEM structure was incomplete and lacked certain residues. To avoid bias from missing residues, we used AlphaFold3 to generate the full native structure to compare with its QTY analog. We did the same with AT5G2, AT5G3, and ATPMK, which all lacked PDB entries. This separation was made to ensure that each QTY analog was compared against the most complete and appropriate native structure.

### Structural superposition and RMSD

Native and QTY protein structures, as well as CryoEM-native and AlphaFold3-native structures when both were available, were superposed in PyMOL (https://pymol.org), and RMSDs were computed from the resulting alignments. We used unrelaxed RMSD values for all reporting. For simplicity and clarity, unstructured loops and extraneous protein monomers were removed from the figures before superposition. For ATP68, the unsegmented superposition was used as the primary analysis. Secondary superpositions of segmented residues 2–20 and 21–42 were also performed to evaluate local structural similarity within the two helical segments.

### Additional structural and membrane-placement analyses

The same native reference structures used in the RMSD analyses were compared with their corresponding QTY structures with US-align (Universal Structural alignment, https://zhanggroup.org/US-align/) to calculate TM-scores and aligned-residue counts [58]. α-Helical content was assessed using DSSP in UCSF ChimeraX [59,60]. For ATP68, these analyses were performed for the full, unsegmented region and separately for the segmented residues 2–20 and 21–42.

Membrane placement was analyzed using PPM 3.0 (https://opm.phar.umich.edu/ppm_server3) with the inner mitochondrial membrane setting, and predicted hydrophobic thickness, tilt, and membrane-transfer free energy (ΔG_transfer_) were reported [61]. PPM analysis was only performed on the full, unsegmented ATP68 model.

For the subunits without complete experimental structure (AT5G1, AT5G2, AT5G3, and ATPMK), the stereochemical quality of the AlphaFold3-native models was assessed using MolProbity (http://molprobity.biochem.duke.edu/), and the MolProbity score, clashscore, Ramachandran statistics, and rotamer outliers were reported [62].

### Protein-protein contact analysis

Native ATP6–c-ring intersubunit contacts were analyzed using Arpeggio (https://biosig.lab.uq.edu.au/arpeggioweb/) with the human ATP synthase CryoEM structure. Analysis focused on QTY-targeted ATP6 residues within 5Å of the c-ring, with ATP6 Arg159 included as an unchanged reference residue due to its mechanistic importance. Arpeggio interaction classifications, including hydrophobic, hydrogen-bonding, ionic, polar, aromatic, and proximal contacts, were analyzed and listed.

### Structure visualization

PyMOL (https://pymol.org) was used to render the superpositions. For ATP68, the primary visualization shows the unsegmented superposition. Because the CryoEM structure showed a clear bend that is not present in the AlphaFold3-predicted models, segment-based analysis was performed and retained as secondary analyses of local helical agreement. UCSF ChimeraX (https://www.rbvi.ucsf.edu/chimerax) was used to render protein models with hydrophobicity patches [60].

## Supporting information

S1 Fig Secondary segment-based structural analyses of ATP68. (A) The CryoEM ATP68 structure (magenta) is aligned and superposed with the segmented AlphaFold3-predicted QTY analog (cyan). Because the native ATP68 CryoEM structure contains a bend not present in the AlphaFold3 predictions, this segment-based superposition was performed using residues 2–20 and 21–42 to assess local helical similarity. The corresponding RMSD values were 1.608 Å and 0.790 Å, respectively. (B) The segmented AlphaFold3-predicted native ATP68 structure (green) is aligned and superposed with the segmented AlphaFold3-predicted QTY analog (cyan) using the same residue ranges. The corresponding RMSD values were 0.955 Å and 0.384 Å, respectively. Two views are shown for each protein (180°). These segmented comparisons are presented as secondary analyses, while the corresponding unsegmented ATP68 comparisons are reported in the main manuscript.
(TIF)

S2 Fig Structural model of ATP68 and local interaction surrounding Tyr21 in the human ATP synthase F0 CryoEM structure. (A) Zoomed-out view showing ATP68 (blue) adjacent to neighboring ATP8 (yellow) and ATP6 (orange), highlighting the bent conformation of ATP68 within the assembled human ATP synthase F0 complex. (B) Enlarged view of the ATP68–ATP8 interface showing ATP68 Tyr21 and ATP8 Gln25 as sticks. The Tyr21 hydroxyl and Gln25 carbonyl oxygen are separated by approximately 2.9 Å, consistent with a possible hydrogen-bonding interaction. ATP6 is shown for structural context.
(TIF)

S3 Fig Mapping of QTY substitutions and native inter-subunit contacts at the ATP6–c-ring interface and key proton-translocation residues. (A) Overall view of ATP6 (orange) and the c-ring (pink) in the assembled human ATP synthase F0 complex. QTY-substituted ATP6 residues located within 5 Å of the c-ring are highlighted in yellow. (B) Close-up of the proton-translocation region showing ATP6 Arg159 (blue) and c-subunit Glu58 (red). Both residues are unchanged by the QTY substitutions and display a side-chain-to-side-chain distance of 2.91 Å. The cGlu58 shown corresponds to chain 2 of the c-ring in the 8H9S CryoEM structure. (C) Arpeggio contact analysis of the 14 QTY-targeted ATP6 residues located within 5 Å of the c-ring. Native c-ring contacts, interaction classifications, and closest interatomic distances are listed. ATP6 Arg159 is included as an unchanged reference residue due to its mechanistic importance; its contact with cGlu58 was classified as hydrogen-bonding, ionic, and polar. All contacts were identified in the native 8H9S structure and therefore do not establish whether the corresponding interactions are retained after QTY substitution.
(TIF)

S4 Fig Membrane topology of eight native ATP synthase F0 subunits. Membrane-topology diagrams are shown for (A) ATP6, (B) ATP8, (C) ATPK, (D) ATP68, (E) ATPMK, (F) AT5G1, (G) AT5G2, and (H) AT5G3. Topological structures were generated using Protter. The membrane is shown in orange and bordered by black lines.
(TIF)

S5 Fig Transmembrane-helix predictions of eight native ATP synthase F0 subunits and their QTY analogs. Predictions are shown for (A) ATP6Native vs ATP6QTY, (B) ATP8Native vs ATP8QTY, (C) ATPKNative vs ATPKQTY, (D) ATP68Native vs ATP68QTY, (E) ATPMKNative vs ATPMKQTY, (F) AT5G1Native vs AT5G1QTY, (G) AT5G2Native vs AT5G2QTY, and (H) AT5G3Native vs AT5G3QTY. Predictions were generated using the Protein Solubility Server (PSS). Red regions indicate predicted transmembrane helices.
(TIF)

S1 Table Additional structural and membrane-placement analyses of the eight native and QTY ATP synthase F0 subunits. TM-score, aligned residues, α-helical content, hydrophobic thickness, tilt, and membrane-transfer free energy (ΔGtransfer) are reported for structural and membrane-placement comparisons. For ATP68, results are shown for the full retained region and separately for residues 2–20 and 21–42 for local analysis. Membrane-placement parameters were calculated only for the full retained region.
(DOCX)

S2 Table MolProbity stereochemical assessment of AlphaFold3-predicted native ATP synthase F0 subunits without complete experimental structures. MolProbity score, clashscore, Ramachandran statistics, and rotamer outliers are reported for AT5G1, AT5G2, AT5G3, and ATPMK.
(DOCX)
