# Supplementary material for: Structure bioinformatics of eight human ATP synthase F0 subunits and their AlphaFold3-predicted reduced-hydrophobicity QTY analogues
Source: PLoS One. 2026 Oct 23;21(10):e0352911. doi: 10.1371/journal.pone.0352911 (PMC13693945; doi:10.1371/journal.pone.0352911)
Supplement: Supplementary file 1 [file pone.0352911.s006.docx]

| **Subunit** | **TM-score** | **Aligned residues/total residues** | **Native α-helix (%)** | **QTY α-helix (%)** | **Native hydrophobic thickness (Å)** | **QTY hydrophobic thickness (Å)** | **Native tilt (°)** | **QTY tilt (°)** | **Native ΔG_transfer_ (kcal/mol)** | **QTY ΔG_transfer_ (kcal/mol)** |
| --- | --- | --- | --- | --- | --- | --- | --- | --- | --- | --- |
| ATP6 | 0.998 | 223/223 | 75.8 | 79.4 | 29.2 ± 1.7 | 8.4 ± 1.1 | 42 ± 2 | 102 ± 2 | −54.3 | −17.2 |
| ATP8 | 0.625 | 26/28 | 85.7 | 92.9 | 27.2 ± 2.0 | 8.2 ± 3.0 | 21 ± 5 | 93 ± 3 | −16.4 | −12.2 |
| ATPK | 0.858 | 59/60 | 78.3 | 75.0 | 28.0 ± 1.7 | 6.3 ± 2.3 | 32 ± 4 | 55 ± 0 | −22.3 | −8.8 |
| ATP68, full retained region | 0.511 | 27/41 | 82.9 | 95.1 | 26.0 ± 0.4 | 9.0 ± 2.6 | 9 ± 4 | 93 ± 0 | −15.3 | −11.0 |
| ATP68, residues 2–20 | 0.332 | 18/19 | 68.4 | 89.5 | — | — | — | — | — | — |
| ATP68, residues 21–42 | 0.575 | 21/22 | 86.4 | 90.9 | — | — | — | — | — | — |
| AT5G1 | 0.989 | 79/79 | 96.2 | 96.2 | 31.0 ± 2.6 | 4.8 ± 0.0 | 41 ± 1 | 81 ± 2 | −25.2 | −14.0 |
| AT5G2 | 0.992 | 79/79 | 94.9 | 94.9 | 28.8 ± 2.3 | 5.8 ± 0.0 | 46 ± 2 | 82 ± 1 | −24.4 | −13.1 |
| AT5G3 | 0.990 | 94/94 | 96.8 | 96.8 | 29.0 ± 2.4 | 3.1 ± 1.1 | 45 ± 1 | 87 ± 2 | −24.2 | −8.0 |
| ATPMK | 0.620 | 22/26 | 76.9 | 88.5 | 26.4 ± 1.5 | 4.7 ± 3.1 | 22 ± 5 | 78 ± 9 | −13.7 | −2.9 |
