# Supplementary material for: Structure bioinformatics of eight human ATP synthase F0 subunits and their AlphaFold3-predicted reduced-hydrophobicity QTY analogues
Source: PLoS One. 2026 Oct 23;21(10):e0352911. doi: 10.1371/journal.pone.0352911 (PMC13693945; doi:10.1371/journal.pone.0352911)
Supplement: Supplementary file 2 [file pone.0352911.s007.docx]

| **Subunit** | **MolProbity score** | **Clashscore** | **Ramachandran favored (%)** | **Ramachandran outliers (%)** | **Rotamer outliers (%)** |
| --- | --- | --- | --- | --- | --- |
| AT5G1 | 0.50 | 0.00 | 100.00 | 0.00 | 0.00 |
| AT5G2 | 1.13 | 1.74 | 100.00 | 0.00 | 1.82 |
| AT5G3 | 1.21 | 4.35 | 100.00 | 0.00 | 0.00 |
| ATPMK | 2.95 | 9.32 | 91.67 | 4.17 | 18.18 |
